# The roles of tertiary lymphoid structures in orchestrating immune responses in peripheral organs

**DOI:** 10.1186/s41232-025-00393-2

**Published:** 2025-09-30

**Authors:** Keisuke Taniguchi, Takahisa Yoshikawa, Motoko Yanagita

**Affiliations:** 1 Department of Nephrology, Graduate School of Medicine, Kyoto University, 54 Shogoin-Kawahara-Cho, Sakyo-Ku, Kyoto, 606-8507 Japan; 2 Institute for the Advanced Study of Human Biology (ASHBi), Kyoto University, Kyoto, Japan

**Keywords:** Tertiary lymphoid structures, Chronic inflammation, Inflammaging, Autoimmune diseases, Cancer immunotherapy

## Abstract

Tertiary lymphoid structures (TLSs) are ectopic lymphoid aggregates that develop in non-lymphoid organs under pathological conditions of chronic inflammation, such as cancer, autoimmune diseases, chronic infections, organ transplantation, and age-related disorders. TLSs produce various cytokines and chemokines, and orchestrate local adaptive immune responses by serving as sites for antigen presentation. TLSs have attracted significant attention because of their multifaceted roles in various diseases. However, the diversity in cellular composition, development, and maturation of TLSs, depending on the disease context and organ, makes it challenging to fully understand their characteristics. Several basic and clinical studies have demonstrated the clinical and pathophysiological roles of TLSs, revealing both their protective and harmful effects. In cancer, TLSs generally activate anticancer immune responses, leading to the suppression of tumor growth. Additionally, they contribute to host defense against pathogens in infectious diseases. Conversely, they can provide a niche for autoantibody production, exacerbating autoimmune diseases and chronic rejection in transplanted organs. In age-related diseases, they may prolong tissue inflammation and hinder tissue repair. The pathophysiological significance of TLSs has prompted the development of therapeutic strategies that target their formation and maturation. However, their potential systemic immunological effects must be carefully considered. Recent advances in single-cell omics technologies have facilitated a deeper understanding of the diverse cellular components of TLSs and their cell–cell interactions, which may contribute to the development of TLS-specific therapies. The fact that TLSs can only be identified using invasive diagnostic methods remains a barrier to further research. Advances in artificial intelligence-driven pathology diagnostics and improvements in imaging technologies for noninvasive detection are expected to accelerate TLS research. Categorizing various conditions with TLSs as 'TLS-related diseases' could deepen our understanding of TLS pathophysiology and lead to the development of novel therapeutic strategies.

## Introduction

In a typical immune response to pathogens, local innate immune cells attempt to control them. Simultaneously, antigen-presenting cells prime lymphocytes in secondary lymphoid organs (SLOs), initiating adaptive immune responses including germinal center (GC) reactions, somatic hypermutation, and class switch recombination [1]. Tertiary lymphoid structures (TLSs), also referred to as tertiary lymphoid tissues, tertiary lymphoid organs, or ectopic lymphoid structures, form in chronically inflamed non-lymphoid organs, such as those affected by malignant tumors, infections, autoimmune diseases, transplanted organs, and age-related disorders [2]. TLSs structurally resemble SLOs in their cellular composition, including diverse immune cells, stromal cells, and vasculature. However, in contrast to SLOs, TLSs lack capsules. Consequently, they are in direct contact with peripheral tissues and function as local hubs of inflammation, facilitating antigen presentation, antibody production, and secretion of pro-inflammatory cytokines [2–4]. Technical advances in single-cell omics have deepened our understanding of the diverse cellular composition of TLSs and their interactions with peripheral tissues, thereby revealing their heterogeneity across organs and diseases [5–10]. 

Various basic and clinical studies have demonstrated the clinical and pathophysiological significance of TLSs, revealing their dual roles as either beneficial or detrimental depending on the disease and organ context. These studies have also explored therapeutic strategies aimed at either TLS resolution or induction as well as their potential utility as prognostic indicators. Among them, TLSs have been identified in multiple organs of aged individuals, suggesting their potential association with age-related alterations in the immune system, termed “immunosenescence” [11–14]. For example, we have demonstrated that TLSs form in the aged kidney after injury and are associated with prolonged inflammation and maladaptive tissue repair, which may serve as potential therapeutic targets for chronic kidney disease (CKD) [6, 7, 15, 16].

This review provides insights into the cellular composition and developmental mechanisms of TLSs and discusses their pathological roles, clinical significance, and potential therapeutic implications in various diseases. Furthermore, we introduce the novel concept of"TLS-related diseases"and offer insights into prospective directions for TLS research.

## Development and maturation of TLSs

**Fig. 1:**
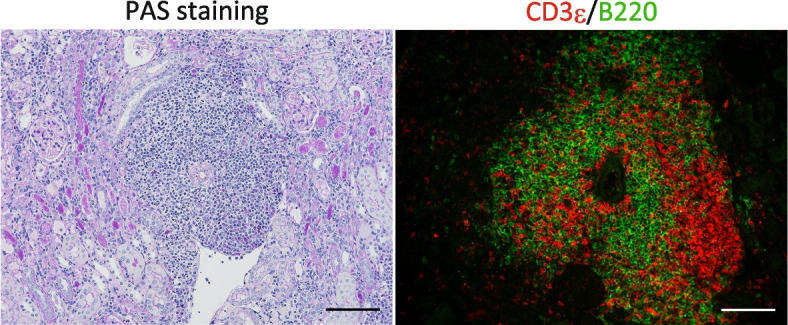
Tertiary lymphoid structures in aged injured murine kidneys revealed by staining with Periodic acid Schiff and immunofluorescence. Histological analysis of 12-month-old murine kidneys 45 days after 30 min of ischemic reperfusion injury by staining with Periodic acid Schiff (left) and immunofluorescence of B220 (green) and CD3ε (red). TLSs are comprised of T and B cells, and develop mainly around the arteries in the kidney. Scale bar = 100 µm

TLSs consist of T, B, and dendritic cells, fibroblasts, lymphatic vessels, and blood vessels, including high endothelial venules (HEVs), resembling SLOs, such as the spleen, lymph nodes, Peyer's patches, and tonsils, in development, components, and structures (Fig. 1) [17, 18]. During SLO development, neuronal cells produce retinoic acid, stimulating mesenchymal cells to secrete chemokine (C-X-C motif) ligand 13 (CXCL13), which then attracts lymphoid tissue inducer cells (LTi) expressing CXCR5 [19]. Lymphotoxin (LT) α1β2 expressed by LTi activates mesenchymal cells expressing LT-β receptor (LTβR) and helps them transdifferentiate into lymphoid tissue organizer cells (LTo) that secrete homeostatic chemokines, such as CXCL13, C–C motif chemokine ligand 19 (CCL19), and CCL21, further attracting LTi and other immune cells. The lymphoid primordium formed by LTi and LTo facilitates HEV differentiation through LTβR signaling. The progressive maturation of HEVs, characterized by the expression of adhesion molecules, such as intercellular adhesion molecule-1 (ICAM-1), vascular cell adhesion molecule 1 (VCAM-1), and mucosal vascular addressin cell adhesion molecule 1 (MAdCAM-1), as well as chemokines, including CCL21, ensures an efficient influx of leukocytes from the bloodstream, contributing to the formation of highly organized lymphoid tissues [20–22].

However, the precise mechanisms underlying TLS initiation and maturation remain unclear. Generally, in chronic inflammation, immune and parenchymal cells, such as epithelial or endothelial cells, activate tissue-resident fibroblasts by producing pro-inflammatory cytokines, such as interleukin (IL)−13, IL-17, and IL-22 [18, 23–25]. These activated fibroblasts that express podoplanin acquire an"immunofibroblast"phenotype [24], which is characterized by the expression of homeostatic chemokines (CCL19, CCL21, and CXCL13), adhesion molecules (ICAM-1 and VCAM-1), and survival factors, such as B cell-activating factor (BAFF) and IL-7 [17], thereby promoting migration, survival, and proliferation of immune cells. Furthermore, T cells within TLSs promote the production of CXCL9, CXCL10, and BAFF in immunofibroblasts via the excessive production of interferon-gamma (IFN-γ) in a signal transducer and activator of transcription 1 (STAT-1) dependent manner, facilitating the recruitment of additional lymphocytes and their survival [6]. These findings indicate that the bidirectional interactions between immunofibroblasts and immune cells play a pivotal role in TLS formation and expansion. During TLS maturation, tissue-resident fibroblasts also differentiate into follicular dendritic cells (FDCs) through stimulation by LTβR and tumor necrosis factor receptor 1 (TNFR1) signaling, developing B cell follicles with help from follicular helper T cells (Tfh), and ultimately forming GCs that produce plasma cells secreting high-affinity class-switched antibodies [11, 26]. Similar to SLOs, HEVs and small blood vessels are also present in TLSs, and play important roles in their maturation and formation [11, 20, 27]. Vascular endothelial cells that lost Notch signaling exhibited HEV-like traits and developed TLSs in various organs, such as kidneys and liver, suggesting that alterations in Notch signaling in endothelial cells might play a crucial role in TLS formation [27].

**Fig. 2:**
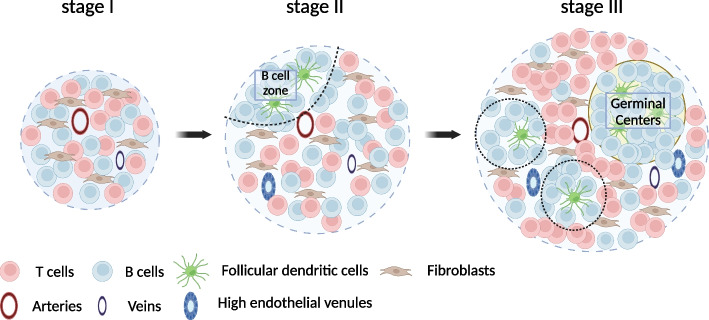
TLS components and staging. Schema of TLS staging. TLSs consist of T, B, and dendritic cells, fibroblasts, blood vessels, and lymphatic vessels. They progress from Stage I to Stage III. Stage I is characterized by unorganized aggregates of proliferating T and B cells. Stage II is defined by the presence of FDCs and B cell zones, but not germinal centers (GCs). Stage III is distinguished by the presence of FDCs and GCs. (Created in BioRender. https://BioRender.com/l28x219)

Depending on the pathological context, TLSs can range from small aggregates of T and B cells to highly organized structures resembling SLOs. This variability leads to inconsistencies in the definition of TLSs and hinders our understanding of their roles, particularly in clinical settings. To address this hurdle, we qualitatively evaluated TLSs by staging them as follows: Stage I, unorganized aggregates composed of proliferating T and B cells; Stage II for those with FDCs but without GCs; and Stage III for those with GCs [16] (Fig. 2). Similar TLS components have been observed in mice and humans, indicating that TLSs mature in a comparable manner across species. This staging method provides evidence supporting its validity. For example, TLSs that form after kidney injury in aged individuals tended to be more mature as the severity of the injury increases. In an ischemia–reperfusion injury model in aged mice, a longer duration of ischemia led to the formation of a greater number of mature TLSs [16]. In aged human kidneys, patients with CKD tended to have more mature TLSs [16]. In kidney transplants, the presence of Stage II TLSs one year after transplantation has been identified as a predictor of poor renal prognosis [28]. A similar approach has been explored in cancer research [29–31]. Notably, patients whose pretreatment biopsy samples contained mature TLSs were significantly more likely to benefit from immune checkpoint inhibitors [29]. These findings suggest that the staging and classification of TLSs significantly improve our understanding of their roles, paving the way for further insights into their implications in various pathological contexts.

## Pathophysiological significance of TLSs

TLSs are observed in various diseases and pathological conditions, including cancers, autoimmune diseases, allograft rejection, infections, and aging [2, 3, 26, 32]. Whether TLSs are beneficial or detrimental to the host depends on the specific context of the organ and disease involved (Table 1). Generally, TLSs exert protective effects against infections and cancers. However, they can be detrimental to autoimmune diseases and aging. The following sections discuss the roles of TLSs in different disease categories.

**Table 1:** Context-dependent roles of TLSs across major pathological settings

Pathological context	Predominant function*	Representative pathological mechanisms	Prognostic impact	Therapeutic implication(s)	Context-specific exceptions
Cancer	Protective	Effector cell activation and Anti-tumor antibody production [40–43]	Larger or mature TLSs is associated with longer overall, progression-free survival and better ICI response [29–31, 33–37, 40]	TLS induction by recombinant CXCL13/CCL21, LTβR agonists, LIGHT agonists, STING agonists, cancer vaccines, and ICIs etc. [35, 44, 45, 47–49, 51–55]	Poor prognosis in HCC, RCC, and irAE-associated TLSs (38.39,56)
Infection	Protective	Effector cell activation and Pathogen-specific antibody production [64–68]	The presence of TLS is associated with better prognosis and effective viral clearance [64–68, 70, 72–74, 76, 77]	TLS induction by live pathogens or vaccines [79–81]	The association between TLSs and fibrosis in SARS-CoV-2 and HCV [12, 82, 83]
Autoimmune diseases	Pathogenic	Autoantibody production [84, 85, 91]	The increase of TLS burden is associated with increased relapse rate and organ damage [84, 85, 87–95]	TLS depletion by BAFF/APRIL blockade or anti-CXCL13 mAbs [97–101]	Certain allergy models show tolerogenic TLSs [96]
Age-related disorders	Pathogenic	Excess local cytokine production (e.g. IFN-γ, TNF-α) [6]	The presence of TLS is associated with worse organ function [13, 16]	TLS depletion by dexamethasone, anti-CD4 mAbs, or CD153–CD30 axis blockade [7, 11, 16]	N/A
Transplantation	Pathogenic OR Protective	･Donor-specific antibody production [122–126] OR ･Niche for Treg or IL-10⁺ B regs [129–134]	Mature TLSs is associated with antibody-mediated rejection, poor graft survival [28, 123–126] OR The presence of TLSs is associated with better graft survival [129–134]	TLS depletion by LTβR blockade or anti-CXCR3/CCL21 mAbs [124, 126, 127] OR N/A	N/A

*Protective,* overall host-beneficial; *Pathogenic,* disease-exacerbating; *TLSs,* tertiary lymphoid structures; *ICIs,* immune-checkpoint inhibitors; *HCC,* hepatocellular carcinoma; *RCC,* renal cell carcinoma; *irAE,* immune-related adverse event; *Treg,* regulatory T cell; *Breg,* regulatory B cell; *HCV,* hepatitis C virus; *CXCL13,* C-X-C motif chemokine ligand 13; *CCL21,* C–C motif chemokine ligand 21; *CXCR3,* C-X-C motif chemokine receptor 3; *LTβR,* lymphotoxin-β receptor; *mAbs,* monoclonal antibodies; *N/A,* not available

### Cancers

The presence of TLSs, especially mature TLSs [29], has been reported to be a favorable prognostic factor in various cancers, such as breast cancer [33], ovarian cancer [34, 35], esophageal squamous cell carcinoma [36], and non-small cell lung cancer [37]. TLSs serve as hubs of anti-tumor immunity in peripheral tissues by activating effector memory helpffver T cells, effector cytotoxic cells, memory B cells, and antigen-producing plasma cells, thereby suppressing cancer growth and metastasis[3]. In contrast, the presence of TLSs is associated with poor prognosis in some cancers, such as hepatocellular carcinoma and renal cell carcinoma, suggesting context-dependent functions of TLSs [38, 39].

In recent years, the association between the efficacy of immune checkpoint inhibitors (ICIs), such as therapies targeting programmed cell death protein 1 (PD-1) and cytotoxic T-lymphocyte-associated protein 4 (CTLA-4), and the presence or localization of TLSs has garnered increasing attention in cancer treatment. ICIs alleviate immunosuppression in the tumor microenvironment and promote TLS formation [3, 29]. In melanoma treated with ICIs, B cells within TLSs proliferate and express *activation-induced cytidine deaminase A* (*AICDA*), a gene involved in class switching and affinity maturation, and the *B cell lymphoma 6* (*BCL6*), which encodes a transcription factor that regulates germinal center formation, demonstrating B cell maturation within TLSs [40]. Additionally, tumors with infiltrating TLSs exhibit a significantly higher frequency of CD8⁺ T cell infiltration within the tumor [40]. Moreover, a high TLS-related gene expression signature has been positively correlated with prolonged overall survival [40]. Similar findings have been observed in other cancers, such as ovarian cancer and renal cell carcinoma, where the presence of TLSs has been correlated with local CD8⁺ T cell activation, B cell infiltration, and improved prognosis [40–43].

Consequently, there is growing attention concerning the induction of TLSs as a potential therapeutic strategy for cancer immunotherapy. Animal experiments have demonstrated that local administration of TLS-related cytokines and chemokines can induce TLSs and improve the prognosis in cancer models. For instance, in ovarian cancer, CXCL13 expression correlates with the presence of TLSs and favorable prognosis. In murine models of ovarian cancer, the exogenous administration of recombinant CXCL13 induced peritumoral TLSs, increased CD8^+^ T cell infiltration, and improved survival rates [35, 44]. Furthermore, gemcitabine combined with local injections of CXCL13 and CCL21 into tumors in a murine model of pancreatic cancer-induced TLSs enhanced CD8^+^ T cell infiltration, decreased FOXP3^+^ regulatory T cells, and reduced tumor size compared to the vehicle-treated group [45]. However, CXCL13 has also been reported to promote tumor metastasis by facilitating the migration of regulatory B cells and the production of IL-10 [46]. Further research is essential to evaluate the efficacy and safety of CXCL13-based treatments. Similarly, LTβR agonists and LIGHT induced HEV and TLS formation, increased lymphocyte filtration, and enhanced the anti-tumor effects of ICI, prolonging the survival period of tumor-bearing mice [47–49]. In the tumor microenvironment, structurally and functionally abnormal blood vessels impair normal angiogenesis and lymphangiogenesis, thereby suppressing anti-tumor immune responses, including TLSs formation [50]. Angiogenesis inhibitors exert anti-tumor effects by restoring this balance through the process of vascular normalization. For instance, in a murine melanoma transplantation model, local administration of a low dose of a STING agonist upregulated expression of angiogenesis-inhibitory factors, such as *Tnfsf15* and *Cxcl10*, and also increased CD31⁺ blood endothelial cells and LYVE-1⁺ lymphatic endothelial cells, indicating normalization of the tumor vascular environment. This process led to the induction of TLSs, increased infiltration of CD8⁺ T cells and CD11c⁺ dendritic cells, tumor regression, and prolonged survival [51]. In addition, the granulocyte–macrophage colony-stimulating factor (GM-CSF)-secreting whole-cell cancer vaccine (GVAX) composed of inactivated cancer cells genetically modified to secrete cytokines and GM-CSF for pancreatic cancer, peptide vaccines for melanoma, toll like receptor agonists for gliomas, and human papillomavirus vaccination for high-grade cervical intraepithelial neoplasia, reportedly induced TLSs and improved prognosis [52–55]. Induction of TLSs through these interventions may have synergistic effects with ICIs, enhancing the efficacy of cancer elimination. However, ICIs can cause immune-related adverse events (irAEs) associated with TLS formation via systemic immune activation [56]. Additionally, CD40 agonists induced TLSs and exhibited anti-tumor effects in some preclinical studies. However, they have also been reported to trigger cytokine release syndrome and immune exhaustion [57–59]. These findings highlight the importance of controlling systemic immune alterations during TLS-induced therapies. To avoid systemic immune activation during TLS induction, tumor-specific drug delivery systems, including vascular-targeting prodrugs, nanocarriers that include liposomes, and tumor-specific antibodies, are expected to be effective [47, 51, 60, 61].

### Infection

One of the most important defense systems against infections is the adaptive immune response. The primary site is SLOs. However, accumulating evidence has demonstrated that TLSs also play a significant role in the defense against infection [62]. Formation of TLSs in the lungs, referred to as induced bronchus-associated lymphoid tissue (iBALT), has been widely reported in respiratory infections [63]. Infection with influenza virus or *Mycobacterium tuberculosis* (Mtb) induces iBALT, which serves as an important immune hub in the lungs for the GC reaction, production of neutralizing antibodies, and activation of CD8^+^ T cells [64–67]. Interestingly, even in the absence of SLOs, mice could still form iBALT and induce B and T cell responses to the influenza virus, enabling them to withstand high viral exposure [64, 65]. These observations indicate that iBALT constitutes a frontline defense mechanism against respiratory pathogens. To substantiate these findings, in young children with insufficiently developed acquired immune systems, iBALT formed in the lungs during respiratory infections contains GC and contributes to B cell differentiation, class switching, and somatic hypermutation, effectively compensating for immature SLOs [68]. IL-1α and Th17 cells are key drivers of iBALT formation during infection [63]. IL-1α, a prototypical alarmin functioning as a damage-associated molecular pattern, is released from necrotic alveolar macrophages and epithelial cells following pulmonary infection [69]. Whereas administration of recombinant IL-1α﻿ to wild-type mice promoted iBALT development in a 10^5^ TCID50 H3N2 influenza-virus infection model, *Il1r1* knockout mice, which lacked IL-1 receptor, failed to generate iBALT, potentially causing prolonged viral infection. *Il1r1* knockout recipients that received wild-type bone marrow lacked iBALT, indicating that IL-1 receptor signaling within the stromal, not hematopoietic cells, was indispensable for iBALT formation [70]. Th17 cells, which secrete IL-17 and IL-22, also contribute to iBALT induction and drive chronic tissue inflammation and organ dysfunction [71–74]. In a model of the highly pathogenic H5N1 influenza virus, IL-17 knockout mice exhibited reduced expression of CXCL13 and CXCR5 in the lungs along with impaired iBALT formation, leading to increased susceptibility to infection [71]. IL-23 plays a crucial role in the maintenance and functional activation of Th17 cells and, in Mtb infection models, IL-23 knockout mice exhibited decreased expression of IL-17 and IL-22, impaired iBALT formation and increased susceptibility to infection [72, 73]. Conversely, iBALT still developed in *Il17a* and *Il17f* double-knockout mice following infection with modified vaccinia virus Ankara, indicating that the requirement for IL-17 in iBALT formation may be context-dependent [75]. While reports of TLSs in infections often focus on iBALT, TLS formation has also been documented in chronic infections of other organs. In patients with chronic HBV infection treated with tenofovir disoproxil fumarate and pegylated interferon alpha, TLSs were observed in the liver tissue at the start of treatment and decreased after therapy [76]. In a model using chimpanzees with chronic hepatitis B virus infection, Toll-like receptor 7 agonist-induced TLSs and TLS formation temporally correlated with the antiviral response [77]. Kidneys surgically removed for pyelonephritis showed TLSs in all cases, frequently located just beneath the renal capsule, at the corticomedullary junction, and around the glomeruli [16]. Additionally, TLSs were frequently observed beneath the urothelium of the renal pelvis in the renal tissues of patients with infectious pyelonephritis. This mechanism was thought to involve urinary IFN-g disrupting the urothelial barrier and prompting interstitial cells beneath the urothelium to produce CXCL9 and CXCL13, thereby inducing TLSs [78].

Additionally, vaccines targeting pathogens can induce TLSs. For example, administration of Bacillus Calmette–Guérin followed by oral exposure to nontuberculous mycobacteria in mice promoted TLS formation in the lungs, leading to enhanced long-term resistance to Mtb [79]. Aerosol vaccination with a sigH mutant of *M. avium subspecies paratuberculosis* also induced TLSs, activated CD4^+^ CCR5^+^ T cells, and improved long-term resistance to Mtb [80]. Additionally, mice exposed to protein cage nanoparticles, self-assembling nanostructures composed of protein subunits that could encapsulate antigens or adjuvants were protected from lethal doses of influenza virus and pneumovirus by inducing iBALT [81]. These findings indicate that TLS induction may be beneficial for defense against infections [63].

Conversely, in severe acute respiratory syndrome-coronavirus-2 (SARS-CoV-2) infection, TLSs may contribute to the persistence of inflammation and fibrosis, leading to long COVID syndrome, which is characterized by symptoms that include shortness of breath and fatigue. Spatial analysis of COVID-19 donor lungs using multiple modalities showed that SARS-CoV-2 infection enhanced CCL21 expression in activated endothelial cells within the adventitial niche surrounding medium-to-large blood vessels and airways, recruiting CCR7^+^ PD1^+^ CD4^+^ T cells and inducing TLSs. The presence of TLSs correlated positively with lung fibrosis [82]. Moreover, a murine model of acute SARS-CoV-2 infection demonstrated TLS development and fibrosis progression in the chronic phase after viral clearance [83]. Additionally, TLSs are observed in the livers of patients infected with the hepatitis C virus and their presence is associated with increased tissue fibrosis and a lower response rate to interferon therapy [12]. In this context, TLSs may damage host organs while engaging in the defense against infections.

### Autoimmune and allergic diseases

TLSs have been identified in a wide range of autoimmune diseases including rheumatoid arthritis [84], systemic lupus erythematosus (SLE) [85], vasculitis [86], IgA nephropathy [87], and IgG4-related diseases [88, 89]. Generally, TLSs are associated with autoantibody production and local immune activation, leading to a poor prognosis in autoimmune diseases. In the synovium of patients with rheumatoid arthritis, GCs are formed within TLSs, leading to somatic hypermutation and class switch recombination, resulting in the production of anti-citrullinated protein antibodies [84]. In lupus nephritis, a strong association has been observed between TLSs and the severity of interstitial damage, as well as the presence of immune complexes in the basement membrane of the renal tubules [85]. Additionally, evaluation of the central nervous system in MRL/MpJ-Fas^lpr/lpr^ mice, a mouse model of lupus, revealed the formation of TLSs containing GCs in the choroid plexus [90], suggesting that local autoantibody production might explain the symptoms observed in seronegative patients with neuropsychiatric SLE. In Sjögren's syndrome, patients with TLSs in their exocrine glands exhibit significantly higher titers of anti-SSA and anti-SSB antibodies, and more severe clinical symptoms [91]. In giant cell arteritis, TLSs around adventitial vasa vasora contained stem cell-like TCF1^+^ CD4^+^ T cells, which had the potential to differentiate into Eomesodermin (EOMES)^+^ cytotoxic T cells and BCL6^+^ T follicular helper-like cells, contributing to treatment resistance [92]. In IgA nephropathy, the presence of TLSs is positively correlated with proteinuria and crescent formation, and more prevalent in the severe group than in the stable group [87].

TLSs also form in allergic diseases, such as allergic conjunctivitis and chronic sinusitis. Analysis of severe allergic conjunctivitis revealed that IL-33 produced by epithelial cells in response to allergen stimulation stimulated ST2^high^ memory Th2 cells, leading to the production of IL-4 and IL-13, which contributed to TLS formation [93]. In chronic sinusitis, TLSs are formed in the nasal polyps and activate effector cells such as mast cells and basophils via local IgE production [94, 95]. These results indicate that TLSs may be pathogenic in allergic diseases. In contrast, TLSs have been reported to contribute to immune regulation in peripheral tissues. iBALT induced by the local administration of lipopolysaccharide delayed the accumulation of Th2 cells after allergen re-exposure, reduced eosinophil infiltration, suppressed GC hyperplasia, and alleviated mucus production [96]. In this context, the authors hypothesized that the sequestration of effector CD4^+^ T cells and Tregs within iBALT limited the diffusion of inflammatory cytokines into the lung parenchyma, thereby suppressing lung inflammation. These results suggest that TLSs may prevent excessive pathology, highlighting their dual roles.

TLSs are generally considered pathogenic in autoimmune diseases and are potential therapeutic targets. Therapeutic interventions targeting the factors critical for the formation and maturation of TLSs have also been investigated. For example, CXCL13 neutralizing antibodies suppressed symptoms and TLS formation in a collagen-induced arthritis model [97]. Additionally, CXCL13 antibody alleviated symptoms in experimental autoimmune encephalomyelitis and mitigated central nervous system symptoms in an MRL/MpJ-Fas^lpr/lpr^ murine model [98, 99]. In the NOD/ShiLtJ mouse model of Sjögren's syndrome, blocking the CD40-CD154 interaction using anti-CD154 antibodies suppressed TLS formation in the salivary glands and decreased the production of autoantibodies [100]. In a model where nucleosome antibodies were passively transferred into AID^⁻/⁻^ MRL/MpJ-Fas^lpr/lpr^ mice, increased BAFF production and TLS formation were observed in the kidneys [101]. Inhibition of BAFF using a soluble BAFF receptor reduced autoantibody levels and proteinuria, alleviated tissue damage, and suppressed TLS formation [101]. A Proliferation-Inducing Ligand (APRIL) shares receptors with BAFF and is involved in B cell maturation and plasma cell maintenance [102]. In human melanoma, APRIL-positive cancer-associated fibroblasts were identified within tumor-associated TLSs, and their proportion positively correlated with the density of B cells in TLSs [103]. Additionally, antibodies against APRIL in a murine model of IgA nephropathy led to improvements in histopathological findings and a reduction in proteinuria [104]. In a Phase 2 trial of an anti-APRIL antibody, sibeprenlimab, in patients with IgA nephropathy, the sibeprenlimab-treated group exhibited significant suppression of serum APRIL levels, reduction in proteinuria, and stabilization of eGFR decline at 12 months compared to the placebo group [105]. The Phase 3 trial(VISIONARY study) is in progress [106]. Although these treatments are not specifically designed to target TLSs, the importance of these molecules in TLS formation suggests that TLSs could be therapeutic targets. However, inhibition of factors required for SLO maintenance may lead to profound immunosuppression. Therefore, the preferred approach is to target pathways specific to TLSs. Development in this area is ongoing [7, 26].

### Aging-dependent chronic inflammation

Age-related changes in the immune system are referred to as “immunosenescence” [107]. Several changes in immune cell types with aging include an increase in autoreactive and exhausted lymphocytes and macrophages with decreased phagocytic capacity, a reduction in natural killer cells, and compromised functionality [107, 108]. Immunosenescence is characterized by increased susceptibility to infections, diminished vaccine responses, increased prevalence of autoimmune diseases, and systemic non-infectious low-grade chronic inflammation, which is known as"inflammaging” [107, 109]. Various mechanisms have been proposed as causes of inflammaging. In aging individuals, the increased production of damage-associated molecular patterns (DAMPs) and decreased clearance efficiency of DAMPs due to impaired phagocyte function lead to the chronic activation of inflammasomes, resulting in chronic inflammation [107]. Database analyses of human blood cells have revealed that aging is associated with increased expression of inflammasome-related genes that include *NLRC4*, *NLRC5*, and *IL-1B* [110]. High expression levels of these genes have been linked to hypertension, atherosclerosis, and increased mortality in older individuals [110]. Serum concentrations of IL-6 and C-reactive protein increase with age, and higher levels correlate positively with lower physical and cognitive abilities, as well as increased mortality risk [111]. Senescent cells acquire a senescence-associated secretory phenotype by secreting inflammatory cytokines and chemokines that induce inflammation in the surrounding environment [112]. In aged mice with a selective ability to eliminate cells expressing the cell senescence marker p16Ink4a (INK-ATTAC mice), the removal of senescent cells suppressed age-related changes and organ dysfunction across multiple organs, suggesting that cellular senescence contributes to various organ dysfunctions through inflammation and impaired tissue repair [113]. Aging alters the gut microbiota, reduces beneficial resident microbes, and leads to microbial dysbiosis, which disrupts immune regulation and contributes to inflammaging [114].

Unsurprisingly, TLSs have been observed in individuals with immunosenescence because both conditions are closely associated with chronic inflammation [107]. For example, TLSs have been observed in aged kidneys [16], livers [12], bladders [13], and lacrimal glands [14]. TLSs formed in aged kidneys could be pathogenic because they could activate adaptive immunity and produce large amounts of cytokines, such as IFN-γ and tumor necrosis factor-alpha, which potentially inhibited normal repair of surrounding renal tubules [6]. The formation of TLSs in the bladder solely due to aging may be linked to increased bladder disorders associated with age, including overactive bladder/urge urinary incontinence, interstitial cystitis/bladder pain syndrome, and recurrent urinary tract infections [13]. TLSs have also been noted in age-related diseases, such as atherosclerosis [115], CKD [11, 16], and chronic obstructive pulmonary disease (COPD) [116]. The frequency and maturation stage of TLSs are higher in elderly patients with CKD than in patients without CKD [16]. In murine cancer models, administration of anti-PD-1 antibodies induced irAEs in the liver, kidneys, and lungs exclusively in aged individuals, leading to damage to these organs [56]. This study demonstrated that in irAE-associated pulmonary TLS formation, CD4⁺ T cells producing IL-21, which increased in numbers with age, played a crucial role in upregulating CXCL13 expression in myeloid cells [56]. The incidence of COPD increases with age and is considered an age-related disease. In COPD, various immune cells have been identified as reactive to self-antigens, and iBALT formation is significantly promoted in the lungs, particularly under severe conditions [116].

Some reports on age-related TLSs have indicated their pathogenic potential. However, research on interventions targeting them remains limited. Previously we have reported that the administration of steroids and anti-CD4 antibodies in models of kidney injury in aged mice resulted in a reduction in TLS size and tissue damage, suggesting that TLSs in aging-related diseases might be potential therapeutic targets [11, 16]. Our single-cell RNA sequencing analysis of CD45^+^ immune cells from TLSs formed in aged kidneys, along with pathological examination, revealed the accumulation of *Tnfsf8* (encoding CD153)^+^ senescence-associated T cells (SATs) and *Tnfrsf8* (encoding CD30)^+^ age-associated B cells (ABCs) within the TLSs. These surface antigens are specific to SATs and ABCs, and ligand-receptor analysis predicts that their interactions occur via the CD153-CD30 signaling pathway. In a kidney injury model of aged CD153 knockout mice, suppression of TLS formation, reduction of ABCs, and mitigation of kidney fibrosis and dysfunction were observed. Similarly, in a kidney injury model of aged CD30 knockout mice, TLS formation was suppressed, with a decrease in the primary functions of SATs, including the production of IL-21 and IL-10, which assisted B cell maturation and proliferation. These findings show that the CD153-CD30 axis is essential for TLS expansion and subsequent kidney injury, and that targeting this axis could be a promising approach for TLS-specific treatment [7]. Murine SATs resemble human peripheral helper T cells (Tph) in terms of B cell helper functions and cell surface antigens[117]. Tphs are PD-1^high^ CXCR5^−^ CD4^+^ T cells that increase in autoimmune diseases such as rheumatoid arthritis and SLE, contributing to disease pathology [118, 119]. Although a pure increase in Tph due to aging has not been reported, Tph increases in autoimmune diseases, where immunosenescence is a potential concern, interacts with ABCs [120], and is involved in TLS formation. These findings suggest that Tph is a potential therapeutic target for TLSs [121].

### Transplanted organs

In organ transplantation, advances in immunosuppressive therapy have significantly reduced the incidence of acute rejection and greatly extended the survival of transplanted organs. However, the incidence of chronic rejection has not changed considerably, which may be partially attributed to TLSs [32]. TLSs are observed in the chronic phase after transplantation across a wide range of organs, including the kidneys, heart, and lungs, and contribute to chronic rejection through the production of antibodies and cytokines directed against the donor organ, worsening the graft prognosis[122]. In the context of cardiac transplantation, TLSs that arise beneath the endocardium, referred to as “Quilty lesions”, have been associated with acute cellular rejection, antibody-mediated rejection, and graft vasculopathy [123]. In a model of intra-abdominally transplanted vascularized cardiac allografts used to assess rejection, TLSs that formed within the graft contained antibody-secreting cells, implying that these structures drove donor-specific antibody production [124]. A similar pattern was observed in kidney transplantation. TLSs within the graft produced donor-specific antibodies and led to increased graft loss [125]. Further evidence was provided by studies using kidneys from rat insulin promoter-lymphotoxin alpha (RIP-LT﻿α) transgenic mice, which developed spontaneous renal TLSs. When these grafts were transplanted into LTβR-deficient recipients lacking SLOs, pre-formed TLSs alone generated donor-specific antibodies and triggered rejection [126]. Our investigation of TLSs in transplanted kidneys that were not rejected yielded intriguing findings. Remarkably, even in the absence of rejection, TLSs formed in half of the patients six months after transplantation, and those who developed mature TLSs with FDCs 12 months after transplantation exhibited poor renal prognosis [28]. Notably, mature TLSs were suppressed in the ABO-incompatible group that received rituximab prior to transplantation compared to the ABO-compatible group without rituximab [28]. These findings highlight TLSs as potential therapeutic targets in transplanted organs. LT﻿α1/β2–LTβR axis and homeostatic chemokines are key pathways driving TLS neogenesis in grafts and are currently under investigation as potential therapeutic targets. In the aforementioned cardiac allografts used to assess rejection, LTβR blockade suppressed TLS formation, prolonged graft survival, and reduced autoantibody levels [124]. In a model where kidneys obtained from B6 wild-type or LTβR knockout mice were transplanted into BALB/c mice, LTβR knockout grafts exhibited prolonged engraftment, reduced immune cell infiltration, and suppressed rejection [126]. In an arterial graft transplantation model that induced atherosclerosis, the administration of neutralizing antibodies against CCL21 and CXCR3 suppressed TLS formation and prevented the progression of atherosclerosis [127]. In contrast, rituximab treatment for chronic antibody-related rejection in kidney transplantation was reported to fail to suppress TLS formation in grafts despite peripheral blood depletion of B cells, with local antibody production persisting [128]. The authors discussed that this outcome might result from the abundance of survival factors, such as BAFF, within TLSs [128].

Conversely, regulatory tertiary lymphoid organs (rTLOs) reportedly exert organ-protective functions in transplanted organs [32, 122]. In a murine model of MHC-mismatched renal transplantation in which DBA/2 mouse kidneys were transplanted into C57BL/6 mice, rTLOs containing numerous regulatory T cells formed around the artery, inducing immune tolerance [129, 130]. Single-cell RNA sequencing analysis revealed that rTLOs may contribute to immune tolerance in allografts by shifting the CD8^+^ T cell phenotype from cytotoxic to exhausted/regulatory and increasing the number of regulatory B cells secreting IL-10 [131, 132]. In addition, iBALT in the lung retransplantation model supports the concept of rTLOs. Acute rejection occurred when lungs from B6 mice were transplanted into non-immunosuppressed CBA mice. However, grafts transplanted into immunosuppressed CBA mice were maintained [133]. Furthermore, the same grafts were re-transplanted into non-immunosuppressed CBA mice, where the graft remained viable [133]. In these lungs, regulatory T cells within iBALT inhibited T cell–B cell interactions, prevented antibody-mediated rejection, and increased lung engraftment rates, indicating that iBALT functions as an rTLO [133, 134]. Thus, heterogeneity of TLSs in transplantation has been suggested, highlighting their diverse roles.

## Conclusions and future prospectives

TLSs are formed in various diseases involving chronic inflammation and have been reported to play context-dependent roles as immune response hubs in peripheral tissues. However, the clinical relevance of the TLSs remains unclear. Currently, TLSs diagnosis relies on detailed pathological examination of tissue samples, making it challenging. Recent advancements in artificial intelligence for pathological and imaging diagnoses may help address some of these limitations. An artificial intelligence based quantitative TLS scoring system in hematoxylin–eosin-stained images of multiple gastrointestinal cancers found that TLS scores were an independent prognostic factor related to survival [135]. A weakly supervised deep-learning model trained on lupus-nephritis biopsy slides predicted 12-month treatment response, and TLSs were observed in the regions associated with non-remission [136]. Radiomics pipelines that integrate histology-based TLS detection with cross-sectional imaging are also under investigation. In the lung adenocarcinoma, a deep-learning model trained on integrating pre-operative CT scans with hematoxylin–eosin-verified TLSs could predict the presence of TLSs from CT data alone and clinical outcomes [137]. Similarly, in hepatocellular carcinoma, machine-learning model trained on MRI scans paired with histologically confirmed TLSs inferred the presence of TLSs directly from MRI data [138]. Collectively, these findings suggest that artificial intelligence-based analysis could identify TLSs without specialized immunostaining and expert annotation, and, in some settings, even without a tissue biopsy. Current studies are constrained by small, single-center datasets and heterogeneous imaging protocols, and standardized methodologies and multi-institutional data aggregation will be crucial for achieving robust, generalizable TLS diagnostics.

Moreover, it is important to develop novel biomarkers. CXCL13 in serum is one of the candidates with reports of correlation with prognosis and activity in autoimmune diseases, but there is still no consensus regarding its association with TLSs [139–141]. The proportion of circulating Tph in peripheral blood correlates with the SLEDAI score and is elevated in patients seropositive for rheumatoid arthritis [118, 119, 121, 142]. Given its association with TLSs, Tph may be a potential predictor of TLS. Imaging targeting markers specific to TLS are also a potentially noninvasive method for diagnosing TLSs. Various imaging technologies targeting the surface antigens of T, B, and dendritic cells have been developed, as well as a new scoring method that integrates findings from contrast-enhanced CT with other clinical parameters. However, sufficient accuracy for diagnosing TLSs has not yet been achieved [143].

Various studies have developed therapies targeting specific pathways or cells associated with TLSs. However, as the cellular components and structures of TLSs are similar to those of SLOs, treatments targeting TLSs may have broad effects on the systemic immune system. Recent advancements in single-cell omics technologies have allowed the simultaneous consideration of interactions among many cells and molecular mechanisms [5, 144]. These techniques may facilitate the development of novel TLS-specific and effective therapies.

**Fig. 3:**
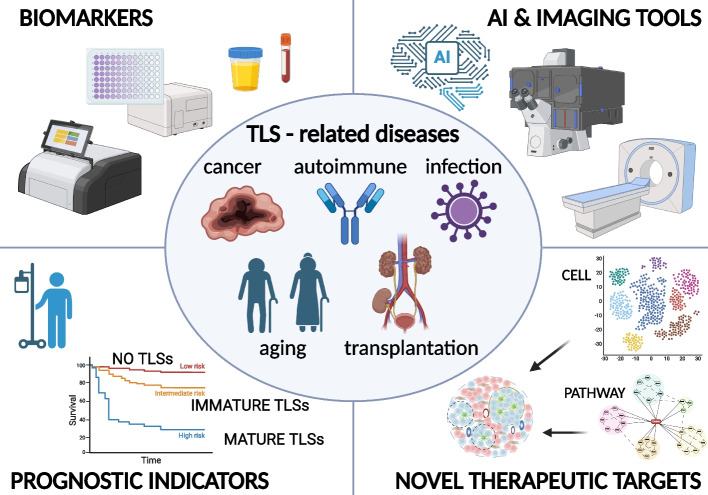
Overview of TLS-related diseases and potential clinical applications. TLSs have been implicated in a wide range of diseases, including cancer, autoimmune diseases, chronic infections, transplanted organs, and age-related disorders. Recognizing these conditions as “TLS-related diseases” based on shared pathophysiological features may facilitate the identification of novel therapeutic targets and clinical applications. The key areas of TLS-related research include (1) discovery of TLS-associated biomarkers, (2) development of artificial intelligence based diagnostic technologies and other noninvasive imaging tools to identify TLSs, (3) potential of TLSs as prognostic indicators, and (4) exploration of TLS-specific molecular pathways for novel therapeutic interventions. Collectively, these efforts aim to establish TLSs as viable therapeutic targets and to improve clinical outcomes in patients with TLS-related diseases. (Created in BioRender. https://BioRender.com/u99k843)

The increasing number of publications focused on TLSs underscores the growing scientific interest in their biological significance and clinical relevance. While TLSs have been associated with a broad range of disorders, most studies have investigated their roles and mechanisms within specific contexts. Considering that TLSs universally function as local immune hubs in chronically inflamed peripheral tissues, we propose the overarching concept of"TLS-related diseases."This concept is defined as conditions wherein the formation of TLSs in affected tissues contributes to disease pathogenesis. A disease is classified as a TLS-related disease when the following two criteria are met: (i) Histological evidence of TLSs—well-organized lymphoid aggregates are visible on hematoxylin–eosin sections, and/or proliferating T- and B-cell clusters are confirmed by immunostaining; (ii) Clinical association—the presence, size, or maturity of TLSs shows an association with disease severity or patient outcome, indicating their roles in pathogenesis. By adopting this framework, diagnostic and therapeutic insights that originally developed in a single disease context may be readily transferred across diverse TLS-related diseases (Fig. 3). For example, TLS-based diagnostic tools developed for autoimmune conditions could be repurposed to predict the presence of TLSs in cancer. Nevertheless, applying this framework requires careful recognition that TLSs may originate through context-specific developmental programs and can exert divergent effects on the host. Despite these complexities, reconceptualizing disparate observations under the unified framework of"TLS-related diseases"could provide a novel conceptual platform for identifying shared therapeutic and diagnostic targets.

## Data Availability

Not applicable.

## Abbreviations

ABCs: Age-associated B cells
AICDA: Activation-induced cytidine deaminase A
APRIL: A proliferation-inducing ligand
BAFF: B cell-activating factor
BCL6: B cell lymphoma 6
CCL19: -C motif chemokine ligand 19
CKD: Chronic kidney disease
COPD: Chronic obstructive pulmonary disease
CTLA-4: Cytotoxic T-lymphocyte-associated protein 4
CXCL13: Chemokine (C-X-C motif) ligand 13
DAMPs: Damage-associated molecular patterns
EOMES: Eomesodermin
FDCs: Follicular dendritic cells
GC: Germinal center
GM-CSF: Granulocyte-macrophage colony-stimulating factor
GVAX: GM-CSF-secreting whole-cell cancer vaccine
HEVs: High endothelial venules
iBALT: Induced bronchus-associated lymphoid tissue
ICAM-1: Intercellular adhesion molecule-1
ICIs: Immune checkpoint inhibitors
IFN-γ: Interferon-gamma
IL: Interleukin
irAEs: Immune-related adverse events
LT: Lymphotoxin
LTβR: Lymphotoxin-β receptor
LTi: Lymphoid tissue inducer cells
LTo: Lymphoid tissue organizer cells
MAdCAM-1: Mucosal vascular addressin cell adhesion molecule 1
Mtb: Mycobacterium tuberculosis
PD-1: Programmed cell death protein 1
rTLOs: Regulatory tertiary lymphoid organs
SARS-CoV-2: Severe acute respiratory syndrome-coronavirus-2
SATs: Senescence-associated T cells
SLE: Systemic lupus erythematosus
SELENA-SLEDAI: Safety of Oestrogens in Lupus Erythematosus National Assessment-SLE Disease Activity Index
SLOs: Secondary lymphoid organs
STAT1: Signal transducer and activator of transcription 1
Tfh: Follicular helper T cells
TLSs: Tertiary lymphoid structures
TNFR1: Tumor necrosis factor receptor 1
Tph: Peripheral helper T cells
VCAM-1: Vascular cell adhesion molecule 1
